# Modulation of Voltage-Gated Ca^2+^ Channels by G Protein-Coupled Receptors in Celiac-Mesenteric Ganglion Neurons of Septic Rats

**DOI:** 10.1371/journal.pone.0125566

**Published:** 2015-05-27

**Authors:** Mohamed Farrag, Lacee J. Laufenberg, Jennifer L. Steiner, Gregory E. Weller, Charles H. Lang, Victor Ruiz-Velasco

**Affiliations:** 1 Department of Anesthesiology, Penn State College of Medicine, Hershey, PA, United States of America; 2 Department of Surgery, Penn State College of Medicine, Hershey, PA, United States of America; 3 Department of Cellular & Molecular Physiology, Penn State College of Medicine, Hershey, PA, United States of America; Indiana University School of Medicine, UNITED STATES

## Abstract

Septic shock, the most severe complication associated with sepsis, is manifested by tissue hypoperfusion due, in part, to cardiovascular and autonomic dysfunction. In many cases, the splanchnic circulation becomes vasoplegic. The celiac-superior mesenteric ganglion (CSMG) sympathetic neurons provide the main autonomic input to these vessels. We used the cecal ligation puncture (CLP) model, which closely mimics the hemodynamic and metabolic disturbances observed in septic patients, to examine the properties and modulation of Ca^2+^ channels by G protein-coupled receptors in acutely dissociated rat CSMG neurons. Voltage-clamp studies 48 hr post-sepsis revealed that the Ca^2+^ current density in CMSG neurons from septic rats was significantly lower than those isolated from sham control rats. This reduction coincided with a significant increase in membrane surface area and a negligible increase in Ca^2+^ current amplitude. Possible explanations for these findings include either cell swelling or neurite outgrowth enhancement of CSMG neurons from septic rats. Additionally, a significant rightward shift of the concentration-response relationship for the norepinephrine (NE)-mediated Ca^2+^ current inhibition was observed in CSMG neurons from septic rats. Testing for the presence of opioid receptor subtypes in CSMG neurons, showed that mu opioid receptors were present in ~70% of CSMG, while NOP opioid receptors were found in all CSMG neurons tested. The pharmacological profile for both opioid receptor subtypes was not significantly affected by sepsis. Further, the Ca^2+^ current modulation by propionate, an agonist for the free fatty acid receptors GPR41 and GPR43, was not altered by sepsis. Overall, our findings suggest that CSMG function is affected by sepsis via changes in cell size and α2-adrenergic receptor-mediated Ca^2+^ channel modulation.

## Introduction

Sepsis and sepsis-related multi-organ failure remain a major challenge in the care of patients in the intensive care unit (ICU) with the mortality rate in patients with sepsis often being double that of critically ill patients without sepsis [1]. Sepsis involves a systemic inflammatory response syndrome (SIRS) and a compensatory anti-inflammatory response syndrome (CARS) [2–3]. SIRS is an aggressive inflammatory state initiated by immune and inflammatory systems characterized by elevated tumor necrosis factor (TNF)-α and several interleukins (ILs) including IL-1 and IL-6. CARS counteracts this inflammatory response by the increased secretion of other cytokines, such as IL-10 and IL-1 receptor antagonist, which limit the overzealous proinflammatory response in an attempt to reestablish homeostasis [2]. An imbalance between these responses can lead to cardiovascular and multi-organ system failure, manifested as septic shock, which is typified by arterial vasodilation and an attenuated pressor response to catecholamines [4].

Sepsis compromises the function of both the immune and autonomic nervous systems as well as their interaction [2,5,6]. Hemodynamic instability observed during sepsis, including the loss of systemic vascular resistance, may result from an imbalance between sympathetic and parasympathetic activity. For instance, spectral analysis of hemodynamic parameters in septic patients showed the presence of autonomic dysfunction contributed to circulatory failure during the early stages of sepsis [7]. Further, chemical ablation of the sympathetic nervous system in mice increased dissemination of Gram-positive bacteria, while decreasing it with Gram-negative bacteria [8]. Finally, administration of lipopolysaccharide (LPS; endotoxin) to healthy volunteers suppressed sympathetic vasomotor tone [9]. These findings suggest that the sympathetic nervous system (SNS) plays a dual role in sepsis, which can be either beneficial or harmful depending on the immune system effector.

Splanchnic circulation is regulated in part by the autonomic nervous system and circulating vasoactive peptides and hormones. Pooling of blood in the splanchnic circulation is another cardiovascular abnormality of sepsis and septic shock as splanchnic blood flow and oxygen consumption increase significantly in septic patients [10]. The superior mesenteric circulation, supplying the splanchnic organs, is predominantly innervated by neurons from the celiac-superior mesenteric ganglion (CSMG). Under inflammatory conditions, various immunomodulatory agents escape through capillary fenestrations to reach the sympathetic nerve terminals and effect neurotransmitter release [11]. It has been shown that sympathetic blockade of the celiac plexus led to hemodynamic instability and increased mortality in a canine model of sepsis [12], suggesting that sympathetic tone of this ganglia is crucial in maintaining homeostasis during sepsis.

Neurotransmitter release is regulated by Ca^2+^ entry via voltage-gated Ca^2+^ channels, especially the N-type Ca^2+^ channel subtype. After neurotransmitters and neuropeptides bind to their cognate G protein-coupled receptor (GPCR), the heterotrimeric G proteins dissociate and the Gβγ dimer binds to N-type Ca^2+^ channels to inhibit Ca^2+^ currents [13]. A number of GPCR have been implicated in the inflammatory response associated with sepsis [5,14–21]. In the present study, cecal ligation puncture (CLP) was performed in rats to test the hypothesis that sepsis would alter the GPCR-mediated modulation of N-type Ca^2+^ channels. CLP is a common model of sepsis that is characterized by abdominal peritonitis and exudation, as well as an increase in the release of proinflammatory cytokines. We evaluated the modulation of Ca^2+^ channels of acutely isolated CSMG neurons from nonseptic and septic rats employing the whole-cell patch-clamp technique.

## Materials and Methods

### Animals

The experiments performed were approved by the Penn State College of Medicine Institutional Animal Care and Use Committee (IACUC). Male, specific pathogen-free Sprague-Dawley rats (Charles River Laboratories, Wilmington, MA) were acclimated for at least seven days with *ad libitum* access to standard rat chow (Teklad Global 2019) and water in a light controlled room (12 hr light/12 dark cycle) prior to induction of sepsis by CLP as described previously [13]. Briefly, the rats were anesthetized with isoflurane (3% induction + 2–3% maintenance; Abbott Laboratories, North Chicago, IL) and the abdomen was shaved and cleaned with betadine. A 2 cm midline laparotomy incision was made and the cecum was then ligated at its base using 4–0 silk. A 20-gauge needle was employed to obtain a through-and-through puncture of the cecum, and the patency of the puncture sites was verified by extruding a small amount of fecal matter. Afterwards, the cecum was returned to the peritoneal cavity and the abdominal wall was sutured with 4–0 silk and the skin was closed using sterile wound clips. The sham control group of rats underwent a midline laparotomy with a closure identical to that of the CLP group. Rats were resuscitated with 10 ml of 0.9% normal saline administered via subcutaneous injection, which was administered immediately after surgery and every 24 hr thereafter. Both sham control and CLP rats were provided water and standard rat chow *ad libitum*. The rats exhibited overt signs of sepsis within 24 hr, including the presence of liquid stool, decreased feeding, cage activity and grooming behavior. The animals were employed for experimentation 48 hr post-sepsis induction, at which time a mortality rate of ~ 20% was observed. Immediately following surgery all rats were injected subcutaneously with 10 ml of warm sterile saline containing buprenorphine (Reckitt Benckiser Pharmaceuticals, Richmond, VA) as well as every 24 hr thereafter for the remainder of the experiment. All animals were monitored every 12 hr for signs of illness or distress including physical appearance (grooming, coat condition, nasal/ocular discharge), inactivity and behavioral responses to external stimuli (e.g. failure to right themselves). Any animals deemed moribund were deeply anesthetized via isoflurane inhalation and the heart was removed to sacrifice the animal.

### Celiac-Superior Mesenteric Ganglion Preparation (CSMG)

CSMG neurons were isolated 48 hr post-sepsis induction. The rats were first anesthetized with CO_2_ and then quickly decapitated with a laboratory guillotine. The CSMG tissue was removed and cleared of connective tissue in ice-cold Hank’s balanced salt solution (Sigma-Aldrich Chemical, St. Louis, MO). The ganglia were enzymatically dissociated in a modified Earle’s balanced salt solution containing 0.6 mg/ml collagenase (Roche Pharmaceuticals, Switzerland), 0.4 mg/ml trypsin (Worthington Biochemical, Lakewood, NJ), and 0.1 mg/ml DNase (Sigma Chemical) in a shaking water bath at 35°C for 60 min. The neurons were dispersed by shaking, centrifuged twice for 6 min at 63 X g, and resuspended in minimal essential medium (MEM), supplemented with 10% fetal calf serum (MidSci, St. Louis, MO), 1% glutamine and 1% penicillin-streptomycin (both from Life Technologies, Carlsbad CA). Finally, the dissociated CSMG neurons were plated onto 35 mm poly-L-lysine coated dishes and stored overnight in a humidified incubator (5% CO_2_/95% air) at 37°C.

### Electrophysiology and Data Analysis

Ca^2+^ channel currents were recorded employing the whole-cell patch-clamp technique. The recording pipettes (Garner Glass Co., Claremont, CA) were generated using a P-97 micropipette puller (Sutter Instrument Co., Novato, CA) and borosilicate glass (King Precision Glass, Claremont, CA). The Ca^2+^ current recordings were made with an Axopatch 200B amplifier (Molecular Devices, Sunnyvale, CA), analog filtered at frequency 2 kHz (–3dB, 4-pole low-pass Bessel filter), and digitized (2–5 kHz) with custom-designed F6 software (written by Stephen R. Ikeda, NIH/NIAAA, Bethesda, MD) equipped with an 18-bit AD converter board (HEKA Instruments, Inc., Bellmore, NY). Both cell membrane capacitance and pipette series resistance were electronically compensated (80–85%).

**Fig 1:**
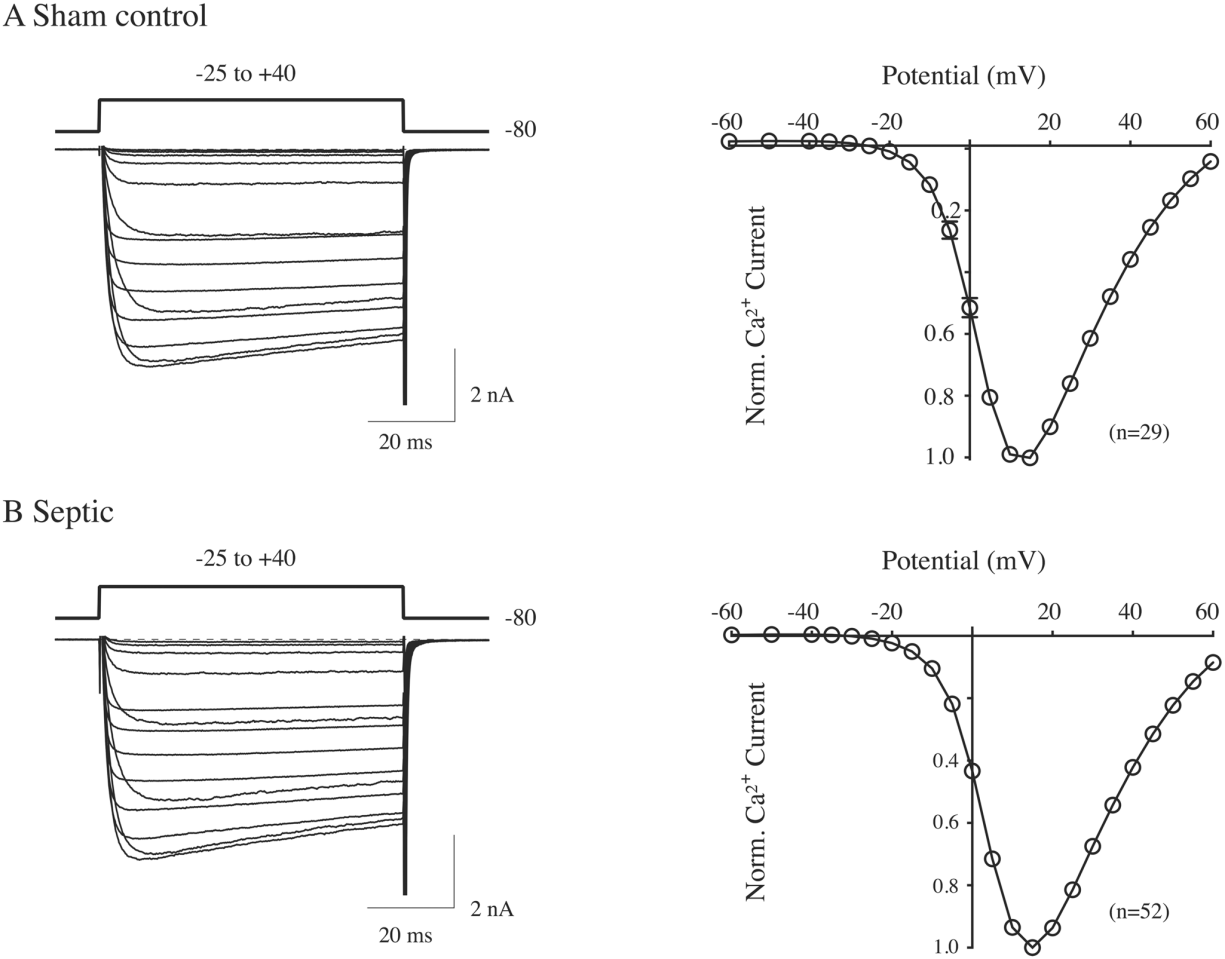
Normalized current-voltage (I-V) relationships of acutely isolated CSMG neurons from sham control (A) and septic (B) rats 48 hr post-sepsis induction by CLP. The I-V curves represent the mean Ca^2+^ current amplitude for each test potential. Ca^2+^ currents were evoked every 3 s with a 70 ms pulse from a holding potential of -80 mV to test potential between -60 and +60 mV. The current amplitude was measured 10 ms following the onset of the test pulse and normalized to +15 mV. The superimposed Ca^2+^ current traces shown to the left were evoked to potentials from -25 to +40 mV. The number of neurons tested for each group is shown in parenthesis.

The Ca^2+^ channel currents of the current-voltage (I-V) relationships (Fig 1) were elicited by a 70 ms depolarizing step to various test pulse potentials from a holding potential of -80 mV. The Ca^2+^ currents illustrated in Figs 3–5 were evoked with the double-pulse voltage protocol (Ikeda, 1991) shown in Fig 1B (top right). The protocol consists of a test pulse to +10 mV (prepulse, ●) followed by a large depolarizing conditioning test pulse to +80 mV, a brief return to -80 mV and followed by a test pulse to +10 mV (postpulse, ○). The peak Ca^2+^ current amplitude was measured isochronally 10 ms after the initiation of the prepulse and postpulse. The agonist-mediated Ca^2+^ current inhibition was calculated as follows: (peak Ca^2+^ current (prepulse before agonist)—peak Ca^2+^ current (prepulse after agonist))/(peak Ca^2+^ current before agonist)) X 100.

Data and statistical analyses were performed with Igor Pro 6.0 (Lake Oswego, OR) and Prism 6.0 (GraphPad Software, Inc., San Diego, CA) software packages with P < 0.05 considered statistically significant. Graphs and current traces were generated with Igor Pro and iDraw (Indeeo, Inc.,) software packages. The concentration-response relationships were determined by the sequential application of the receptor agonist in increasing concentrations. The results were then pooled and the concentration-response curves were fit to the Hill equation. The half-inhibition concentration (EC_50_) values were obtained with Prism 6.0 software (GraphPad Software). All data are presented as mean ± SE.

### Recording Solutions and Drugs

The Ca^2+^ current recording solution (bath) contained (in mM): tetraethylammonium hydroxide (TEA-OH) 145, methanesulphonic acid (CH_3_SO_3_H) 140, HEPES 10, glucose 15, CaCl_2_ 10 and TTX 0.0003, to pH 7.40 with TEA-OH. The pipette solution (internal) contained (in mM): N-methyl-D-glucamine (NMG) 80, TEA-OH 25, EGTA 11, HEPES 10, CaCl_2_ 1, CsCl 20, CsOH 40, MgATP 4, Na_2_GTP 0.3 and creatine phosphate 14, to pH 7.2 with CH_3_SO_3_H. Stock solutions of NE, [d-Ala2-N-Me-Phe4-Glycol5]-enkephalin (DAMGO), propionate (all from Sigma-Aldrich), and nociceptin (Noc, Tocris Cookson, Ellisville, MO) were prepared in H_2_O and diluted in the external solution to their final concentration prior to use. The agonists were applied to the neuron under study with a custom-designed gravity-fed perfusion system that was positioned approximately 100 μm from the cell. In one set of experiments, the CSMG neurons were pretreated with pertussis toxin (PTX, List Biological Laboratories, Campbell, CA) overnight (12–16 hr). PTX was added to the culture medium at a final concentration of 500 ng/ml. At this concentration, PTX has been shown to selectively uncouple Gα_i/o_ G proteins from GPCR [22].

## Results

### Properties of voltage-gated Ca^2+^ currents of CSMG neurons

The purpose of this set of experiments was to compare the voltage-gated Ca^2+^ current properties of CSMG neurons isolated from sham control and septic rats. The I-V relationships for peak Ca^2+^ currents of CSMG neurons from both sham control and septic rats are shown in Fig 1A and 1B, respectively. The superimposed Ca^2+^ currents shown to the right were elicited with depolarizing pulses ranging from -25 to +40 mV. The peak current amplitude was measured isochronally 10 ms from the start of each test pulse. The I-V plots for both groups of rats revealed that the inward current activated at potentials more positive than -20 mV and reached a maximum close to +15 mV. These results indicate the peak Ca^2+^ current did not differ between the two groups of neurons and that sepsis did not overtly affect the I-V relationship of Ca^2+^ channels.

**Fig 2:**
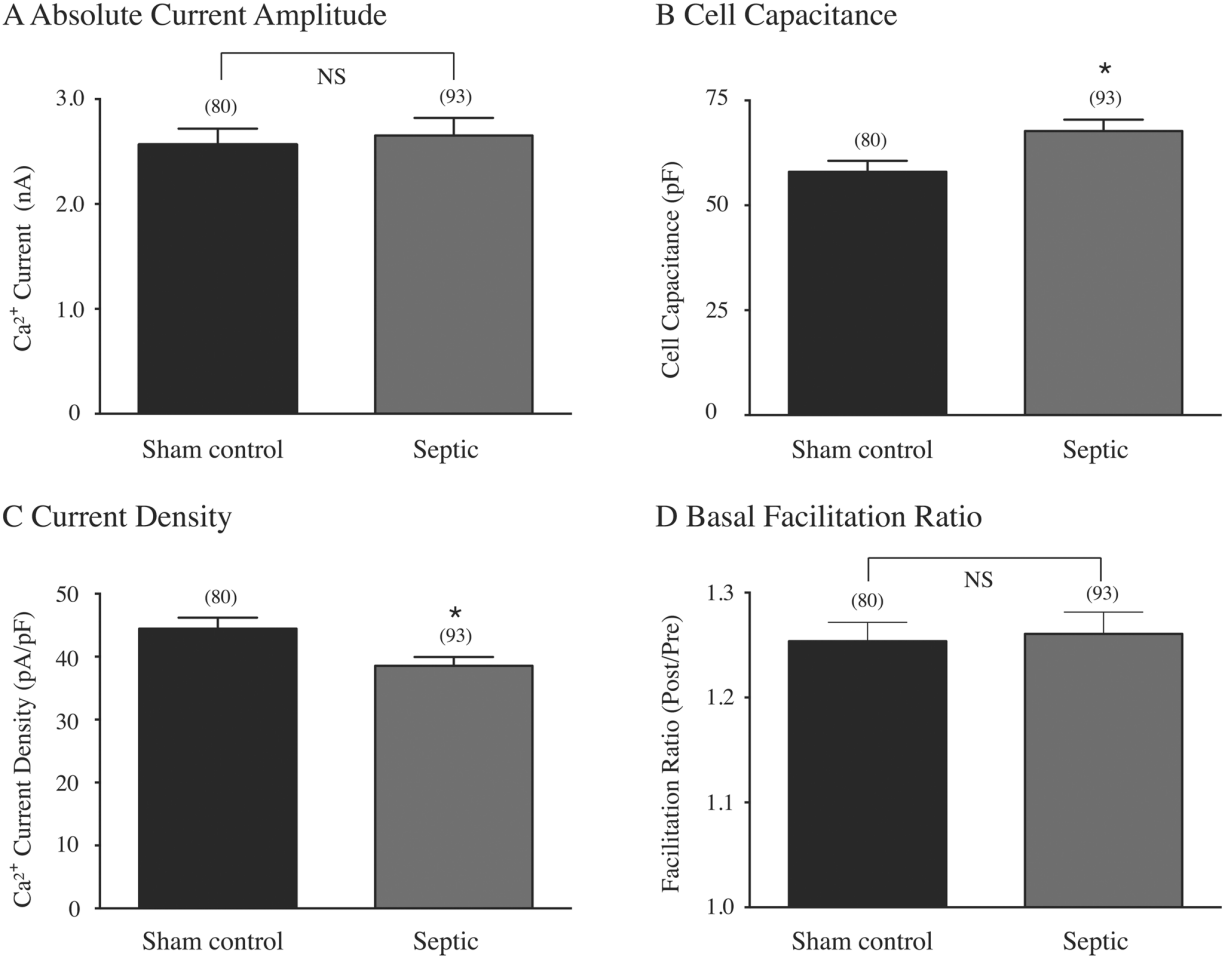
Biophysical properties of CSMG neurons from sham control and septic rats. Measurement of the mean absolute Ca^2+^ current amplitude (A) at the depolarizing potential to +10 mV. Ca^2+^ current density (B) was calculated from the peak Ca^2+^ current amplitude at the test pulse of +10 mV and normalized to membrane capacitance. The cell membrane capacitance (C) was determined from the numerical integration of a transient elicited with a depolarizing pulse from -80 mV to -70 mV prior to electronic compensation. Summary graph of the basal facilitation ratio (D), calculated as the ratio of Ca^2+^ current amplitude determined from the test pulse (+10 mV) occurring after (postpulse) and before (prepulse) the +80 mV conditioning pulse (see Fig 3A, top right). The current amplitude was measured isochronally 10 ms after initiation of the test pulse. The numbers in parenthesis indicate the number of neurons tested; * indicates P < 0.05, unpaired *t*-test, NS indicates not significantly different, P > 0.05.

Fig 2A is a summary plot of the absolute Ca^2+^ current amplitude obtained from both groups of neurons. The results show that the current amplitude was slightly greater (P = 0.70) in CSMG neurons isolated from septic rats compared to neurons from sham control rats. On the other hand, the plot in Fig 2B indicates that the neuron capacitance, a measure of surface area, was significantly (P < 0.05) higher in neurons from septic rats. As a result, the Ca^2+^ current density was significantly (P < 0.05) reduced compared to neurons from sham control rats (Fig 2C).

### Sepsis and the modulation of Ca^2+^ currents by GPCR in CSMG

**Fig 3:**
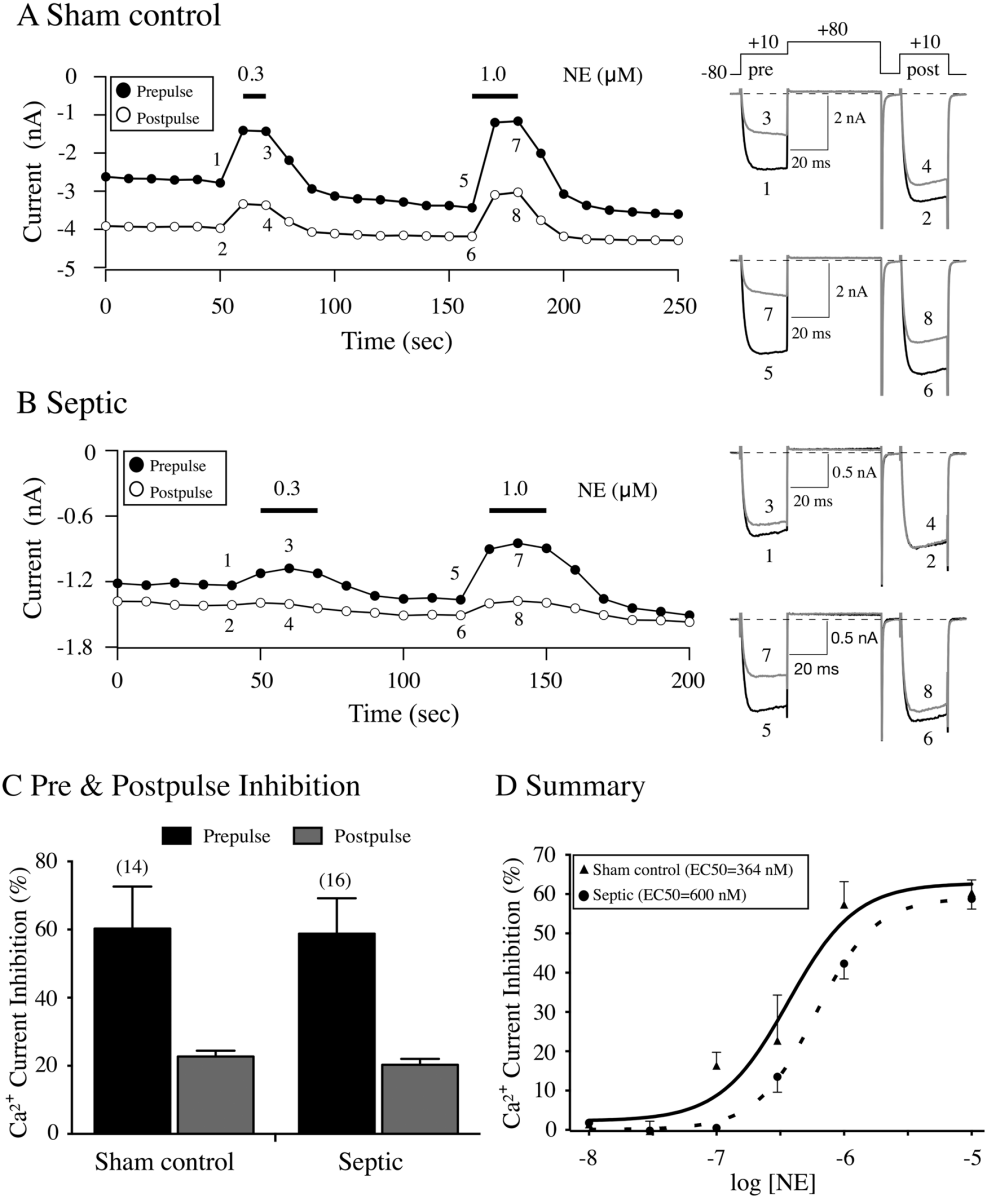
Time courses of Ca^2+^ current amplitude for prepulse (●) and postpulse (○) acquired from the application of NE in acutely isolated CSMG neurons from sham control (A) and septic (B) rats 48 hr post-sepsis induction by CLP. Ca^2+^ channel currents were evoked every 10 sec with the ‘double-pulse’ voltage paradigm (A, top right). The numbered current traces in A and B are shown to the right. C, summary bar graph illustrating the mean (± SE) prepulse inhibition (first test pulse to +10 mV) and postpulse inhibition (second test pulse to +10 mV) mediated by 10 μM NE. The numbers in parenthesis indicate the number of neurons tested. D, NE concentration-response relationship of CSMG neurons from sham control (▲) and septic (●) rats. Each data point represents the mean (± SE) NE-mediated prepulse current inhibition from 4 to 16 neurons, except for 1 and 30 nM where n = 1. The smooth curves were obtained by fitting the points to the Hill equation and the EC_**50**_ (nM) values, shown in the legend, were significantly (P < 0.001) different from each other.

The next set of experiments examined whether sepsis altered the coupling of Ca^2+^ channels with a number of GPCR. Fig 3A shows a time course of peak Ca^2+^ currents and the effect of NE exposure in a CSMG neuron isolated from a sham control rat. The currents were evoked with the double-pulse protocol and the corresponding traces are shown to the right in Fig 3A. Prior to application of the adrenergic receptor agonist, NE (0.3 μM), it can be seen that the activation phase of the prepulse current (trace 1 Fig 3A, right) was fast and a plateau was reached within 5 ms after the onset of the depolarizing pulse. Following exposure to NE, the prepulse current (trace 3 Fig 3A, right) was blocked by approximately 50% and the rising phase of the prepulse exhibited kinetic slowing. This latter property is believed to occur as a result of a voltage-dependent (VD) relief of the block during the test pulse [23]. The facilitation ratio, obtained by dividing the postpulse current by the prepulse current amplitude, is a parameter employed to measure agonist-mediated VD inhibition. That is, before NE exposure (traces 1–2), the conditioning pulse to +80 mV exerted a minor effect on the postpulse current amplitude and the facilitation ratio was ~ 1.5. In contrast, application to 0.3 μM NE led to a greater block of the prepulse when compared to the postpulse (cf. traces 3 and 4) and thus a ‘relief’ of the NE-mediated block by the conditioning pulse and an elevated facilitation ratio of 2.0. After the removal of NE, the currents were allowed to recover and the neurons were next exposed to 1.0 μM NE which blocked the prepulse current (trace 7) almost 70% (cf traces 5 and 7). The facilitation ratios before and during NE exposure were 1.4 and 2.4, respectively.

Fig 3B depicts the time course of the prepulse and postpulse currents recorded from a CSMG neuron isolated from a septic rat and the corresponding numbered traces are shown to the right. When the neuron was exposed to NE (0.3 μM), the prepulse current was inhibited by ~ 15% (cf. traces 1 and 3) and the facilitation ratios prior and during agonist exposure were 1.1 and 1.3, respectively. Following a recovery period, the neuron was exposed to 1.0 μM NE and the prepulse current was inhibited by ~ 38% (cf. traces 5 and 7). The facilitation ratios were 1.1 and 1.6 before and during NE application, respectively.

Fig 3C illustrates the mean Ca^2+^ current inhibition (± SE) of the prepulse and postpulse currents produced by 10 μM NE (the highest concentration employed) in sham control and septic CSMG neurons. The plot indicates that in the presence of a maximal NE concentration, the degree of inhibition of the prepulse (60% control vs. 58%, septic) and postpulse (22%, control vs. 20%, septic) currents in both groups of neurons was similar. Finally, the current inhibition was more potent for the prepulse current than the postpulse current, revealing the VD block by NE (Fig 3C). Fig 2D shows a plot comparing the mean basal facilitation ratio, an index of tonic G protein activation [23]. A statistical comparison of the ratios from both groups of CSMG neurons indicated they did not differ (P = 0.82). Thus, sepsis does not appear to influence tonic G protein activation of CSMG neurons. Fig 3D shows the NE concentration-response relationship for both sham control and septic rats. The data points for each group were fit to the Hill equation. A comparison between both plots indicated that there was a rightward shift of the NE concentration-response relationship and a significantly higher (P < 0.001) EC_50_ (nM) value in CSMG neurons from septic rats when compared to neurons from sham control rats. On the other hand, there was no significant (P > 0.05) difference between % maximum current inhibition (± SE) in sham control and septic neurons (62.8±8.1 and 59.0±1.9, respectively). Although NE was more potent in sham control rats, the agonist exhibited a similar efficacy in both groups of neurons. The Hill coefficient (± SE) values obtained were 1.64±0.89 and 1.90±0.22, and, respectively (P > 0.05).

**Fig 4:**
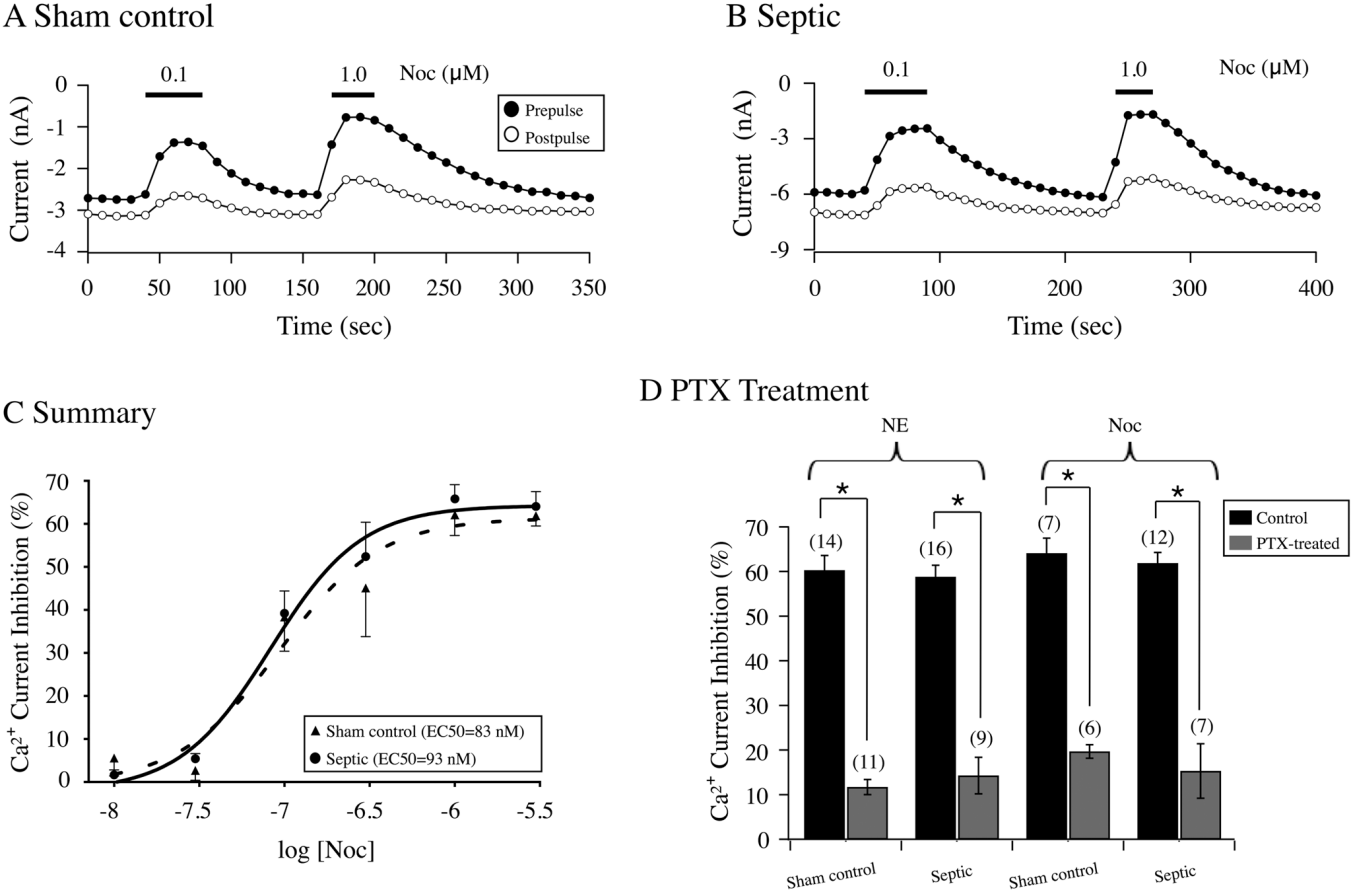
Time courses of Ca^2+^ current amplitude for prepulse (●) and postpulse (○) acquired from the application of Noc in acutely isolated CSMG neurons from sham control (A) and septic (B) rats 48 hr post-sepsis induction by CLP. Ca^2+^ channel currents were evoked as described for Fig 3. C, Noc concentration-response relationship of CSMG neurons from sham control (▲) and septic (●) rats. Each data point represents the mean (± SE) Noc-mediated prepulse current inhibition from 4 to 12 neurons. Both smooth curves were obtained by fitting the points with the Hill equation and the fits were not significantly different (P = 0.95). The legend indicates the EC_**50**_ values (nM). D, effect of overnight pretreatment with PTX (500 ng/ml) on the NE (10 μM)- and Noc (3 μM)—mediated Ca^2+^ current inhibition in both sham control and septic rats. Plot indicates the mean (± SE) current inhibition produced by NE and Noc. Numbers in parenthesis indicate the number of neurons tested. * indicates P < 0.05 compared to each respective control, unpaired *t*-test.

Recent studies have reported that the nociceptin/orphanin FQ peptide (NOP) opioid receptor and its endogenous agonist, Noc, are involved in sepsis and the observed inflammatory responses [16,17]. Thus, we next examined the Noc concentration-response relationships for both groups of CSMG neurons. Fig 4 shows the time courses of peak Ca^2+^ currents for CSMG neurons from sham control (4A) and septic (4B) rats before and after the sequential application of 0.1 and 1.0 μM Noc. In both sham control and septic neurons, Noc (0.1 μM) application inhibited the prepulse currents by 50% (cf. Fig 4A and 4B). After recovery of the Ca^2+^ current amplitude, exposure to 1 μM Noc decreased prepulse current in CSMG neurons from both groups by more than 70%. The results of the Noc concentration-response relationships are plotted in Fig 4C. The estimated values obtained for EC_50_ (nM), Hill coefficient and % maximum current inhibition were 86 and 94 nM, 1.78±0.43 and 1.50±0.60, and 63.8±3.1 and 61.2±5.9, respectively for sham and septic rats. A comparison of these parameters in both groups of neurons indicated that they were not significantly different (P > 0.05).

In a separate set of experiments, both groups of CSMG neurons were pretreated overnight with pertussis toxin (PTX) to examine whether sepsis would alter the PTX-sensitive G proteins (i.e. Gα_i/o_ subfamily) used by adrenergic and NOP receptors to couple with Ca^2+^ channels. Fig 4D is a summary plot illustrating PTX treatment significantly (P < 0.05) disrupted the coupling between adrenergic and NOP receptors with Ca^2+^ channels following application of NE (10 μM) and Noc (3 μM) in CSMG neurons from sham control and septic rats. These results indicate that sepsis does not overtly change the Gα_i/o_ protein signal transduction involved in Ca^2+^ channel modulation.

**Fig 5:**
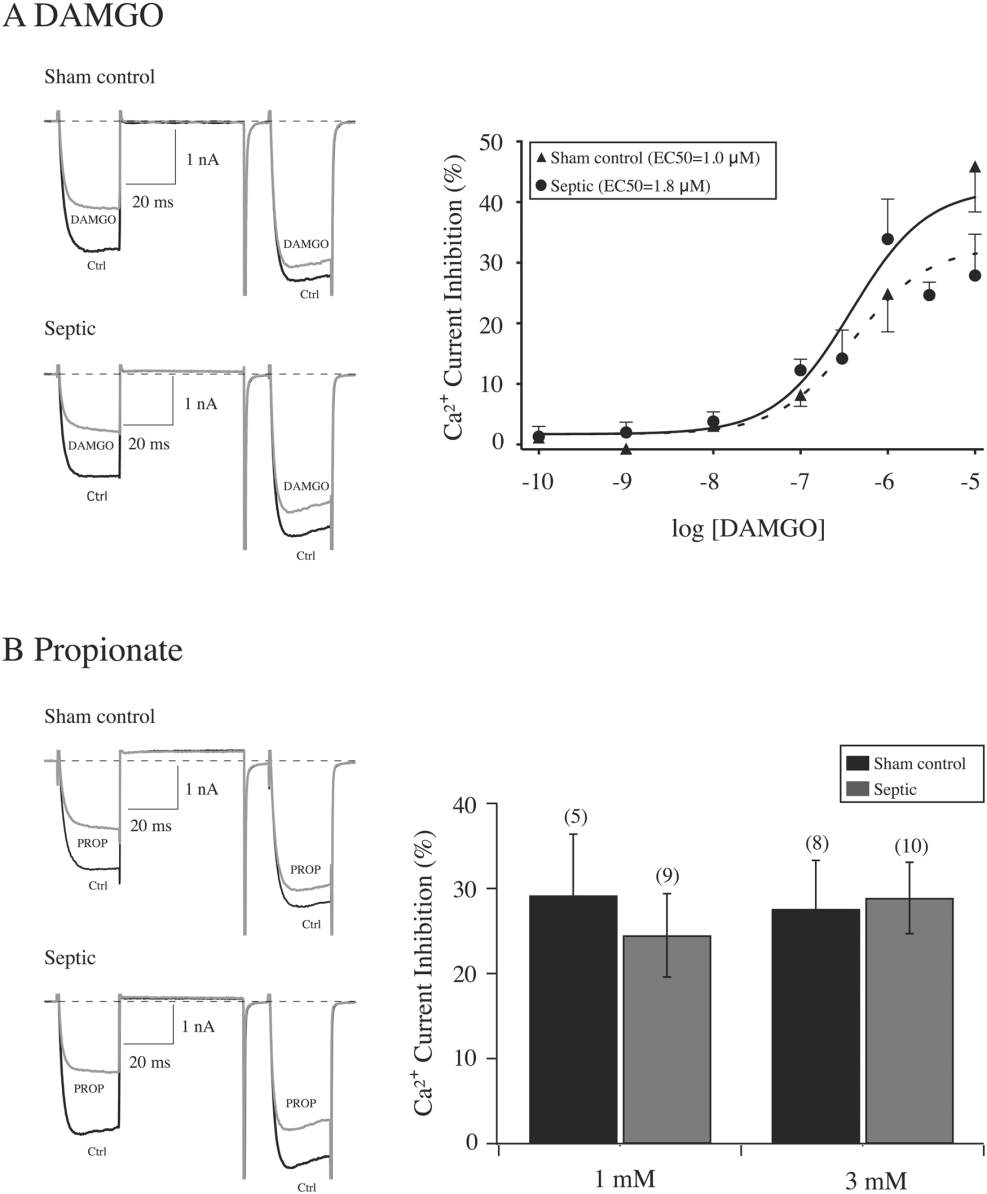
CSMG neurons express mu opioid (A) and free fatty acid (B) receptors. A, left, superimposed Ca^2+^ current traces (evoked as described for Fig 3) in the absence (lower traces, black) and presence (upper traces, gray) of 10 μM DAMGO for sham control and septic rats. A, right, DAMGO concentration-response relationship of CSMG neurons from sham control (▲) and septic (●) rats. Each data point represents the mean (± SE) DAMGO-mediated prepulse current inhibition from 3 to 9 neurons, (except for 0.1 nM DAMGO for sham group where n = 2). Both smooth curves were obtained by fitting the points with the Hill equation and the fits were not significantly different (P > 0.05). The legend indicates the EC_**50**_ values (μM). B, left, propionate—mediated Ca^2+^ current inhibition in sham control and septic rats. B, right, summary plot indicates the mean (± SE) current inhibition produced by 1 and 3 mM propionate. Numbers in parenthesis indicate the number of neurons tested.

In addition to Noc, endogenous levels of other opioid peptides, including β-endorphins, are known to increase during sepsis [18,19]. Thus, the aim of the next set of experiments was to determine whether CSMG neurons also express mu opioid receptors (MOR) and whether sepsis would alter the pharmacological profile of the high affinity receptor ligand, DAMGO. Fig 5A shows the effect of DAMGO (10 μM) application on Ca^2+^ currents in both sham control and septic CSMG neurons. The DAMGO concentration-response relationship shown to the right indicated a tendency for a slightly lower maximal % current inhibition (± SE) in septic neurons (30.0 ± 3.6) than sham control (50.0 ± 2.8; P = 0.09). A comparison of EC_50_ (μM) in both groups of neurons indicated that this parameter did not differ significantly. However, in both sham control and septic groups, 30% of CSMG neurons tested did not exhibit coupling of MOR to Ca^2+^ channels. Therefore, the DAMGO pharmacological profile did not appear to be significantly altered by sepsis.

The short chain fatty acids (SCFA), such as propionate and acetate, are bacterial byproducts in the intestine that activate the GPCR, GPR41 and GPR43 [19,20]. Recently, it was shown that GPR41 and GPR43 transcripts are present in rat CSMG and activation of the former receptor with propionate inhibits Ca^2+^ channel currents in a VD manner [24]. Because the CLP model involves bacterial invasion of the peritoneal cavity, we also examined whether sepsis altered GPR41- or GPR43-mediated modulation of Ca^2+^ channels. Fig 5B (left) shows Ca^2+^ current traces from both control and septic neurons before and during exposure to propionate (3 mM). Similar to the agonists described above, exposure to propionate resulted in VD inhibition of Ca^2+^ currents in both groups of neurons. Under our recording conditions, approximately 50% of sham control and septic CSMG neurons exhibited an apparent response to propionate and the responses varied widely making it difficult to obtain concentration-response relationships. We obtained reliable inhibition of Ca^2+^ currents with 1 and 3 mM propionate. The summary plot shown in Fig 5B shows that at least with either concentration, the propionate-mediated modulation of Ca^2+^ channels was similar for both groups of CSMG neurons.

## Discussion

During sepsis, the vascular tone of the mesenteric circulation is compromised, partly as a result of an altered sympathetic regulation of these vessels. Eventually, as septic shock progresses, unresponsive hypotension occurs in part from autonomic dysfunction. In the present study, the CLP model of peritonitis was employed to determine whether adrenergic-, opioid- and SCFA-mediated modulation of Ca^2+^ channels in CSMG sympathetic neurons would be altered by sepsis. Previous work indicates that N-type Ca^2+^ channels contribute approximately 75% of the macroscopic Ca^2+^ current in CSMG neurons, while the remainder is contributed by L-type channel subtype [11,25,26]. In the present study, no overt changes were observed for the I-V curves or peak command potential (+15 mV) in neurons from either group. However, our results show that sepsis led to a significant decrease of the Ca^2+^ current density of CSMG neurons compared to control rats. The reduction in current density in this group of neurons resulted primarily from a significant increase in membrane capacitance (i.e. surface area) coupled with a modest increase in absolute Ca^2+^ current amplitude. Unlike our findings, a recent report found that the Ca^2+^ current density in mice CSMG neurons was unaffected 6 and 12 hr post-CLP induction [27]. The different species or the time of recordings or both may account for the observed differences. However, decreases in Ca^2+^ current density have been observed in CSMG neurons in a mouse model of the inflammatory disease, colitis [11].

There are a number of possible explanations for the decrease in cell current density. One reason for the sepsis-induced increase in cell surface area could be cell swelling. A number of sepsis studies have reported this effect in skeletal muscle [28], cardiac myocytes [29,30], endothelial cells [31,32], alveoli [33] and renal cortical tubules [34]. The exposure of CSMG neurons to the inflammatory agents released within the peritoneal cavity (i.e. TNF-α and interleukins) may have caused these structural changes. A second possible explanation may have been that neurite outgrowth was enhanced in neurons from septic rats. One study found that co-culturing superior cervical sympathetic ganglia with lymphoid tissue promoted neurite outgrowth [35]. The enhanced neurogenesis was a result of secreted IL-1 and nerve growth factor by the lymphoid tissue. Additionally, exposure to exogenous interleukins IL-3 and IL-6, both released during an inflammatory response, increased neurite outgrowth formation [35]. Third, it is possible that sepsis may have altered the ability of CSMG neurons from septic rats to undergo basal neurite formation following cell isolation. Fourth, small neurons within the CSMG ganglia could have been more susceptible to sepsis and, thus, were not represented in this group of cells. Finally, it is noteworthy that the observed capacitance for both groups of neurons obtained in this study (~ 54 pF) were higher than those previously described [25,36]. The difference between these studies and our study is that the Ca^2+^ currents were obtained within 2–6 hr after cell isolation in the former, while our current recordings were performed following overnight incubation (~ 12–16 hr) of isolated neurons.

One hallmark of sepsis is a considerable increase of both sympathetic nerve activity and plasma levels of NE and epinephrine [2,4,5,37]. The release of NE, the principal neurotransmitter at sympathetic postganglionic nerve terminals, is regulated by α2-adrenergic receptors (i.e. autoreceptor). Our results show that the NE pharmacological profile of CSMG from septic rats was altered 48 hr post-sepsis induction. We observed a rightward shift of the concentration-response curve, consistent with a decrease in NE potency. Thus, CSMG neurons appear to employ a compensatory response that serves to increase NE levels via attenuation of the α2-AR at the nerve terminals. Further studies examining the impact of sepsis on vascular contractile function in response to an increased sympathetic nerve activity are required to ascertain this possibility. A potential limitation of our study is that the electrophysiological recordings were obtained at the soma and not at the nerve terminal, the main site of neurotransmitter release. However, several studies have shown a high correlation of inhibition of transmitter release with Ca^2+^ influx inhibition [38].

Recent clinical and animal studies have shown a link between the NOP receptor signaling system and sepsis [39,40,41] as septic patients have elevated plasma Noc levels which correlate with the severity of the inflammatory responses. We previously found no difference in the modulation of Ca^2+^ channels by Noc in stellate ganglion (SG) of sham and septic rats [13]. We did not find differences in the Noc pharmacological profiles 24 and 72 hr post-sepsis induction. However, we did observe a significant increase in the Noc mRNA transcript, prepronociceptin [14]. Presently, the Noc concentration-response relationship and Noc EC_50_ values were similar for both groups of CSMG neurons. These results suggest that although the NOP receptor:Noc signaling pathway plays a role in sepsis, the signal transduction events modulating N-type Ca^2+^ do not appear to be affected in sympathetic neurons in the CLP sepsis model.

Little information is available regarding sepsis and its effect on heterotrimeric G proteins. Expression levels of the PTX-sensitive Gα_i2_ and Gα_i3_ G proteins have been reported to increase in rat liver plasma membranes 18 hr post-sepsis (CLP) induction [42]. Additionally, CLP-induced sepsis in Gα_i2_ knock-out mice decreased survival rate and increased the production of proinflammatory agents [43]. In our study, CSMG neurons were pretreated with PTX to uncouple Gα_i/o_ proteins from adrenergic and NOP receptors. We posited that an altered expression of nonPTX-sensitive Gα protein subtypes would potentially result in coupling of the receptors to N-type Ca^2+^ channels. However, our results showed that PTX pretreatment significantly and equally abolished the modulation of Ca^2+^ channels in both groups of CSMG neurons. These data suggest that, at least for CSMG neurons, sepsis does not affect coupling specificity of adrenergic and NOP receptors.

Given that expression of MOR has been detected primarily in central and sensory nervous systems, an unexpected finding was that a subpopulation (~70%) of CSMG neurons expressed MOR. Endogenous opioid levels have been reported to be higher in septic patients compared to nonseptic patients [18]. Although opiates exert primarily analgesic effects, intravenous administration of these agents can decrease blood pressure and heart rate [44]. In isolated rat small mesenteric arteries, for instance, morphine exposure causes vessel relaxation [45]. Also, administration of the MOR antagonist, naloxone, prevents endotoxin-mediated hypotension in rats [46]. Thus, the MOR-mediated inhibition of N-type Ca^2+^ channels in CSMG neurons suggests that these receptors modulate neurotransmitter release at nerve endings. Coupled with a weakened vascular response to vasopressor agents in sepsis, it appears that opioid administration would lead to further vasodilation in a number of abdominal organs. Thus, our observations suggest that the clinical use of opiates to minimize pain should be done with caution.

Research interest in GPR41 and GPR43 receptors has been growing to better understand their role in the gastrointestinal tract and the sympathetic nervous system in appetite [47], obesity [48,49,50] and diabetes [51]. Also, one study examined the role of GPR43 expression by polymorphonuclear leucocytes during an inflammatory response following induction of dextrane sodium sulfate-induced colitis [21]. It was revealed that during acute colitis, GPR43 KO mice had a lower invasion of polymorphonuclear leucocytes and an increased mortality resulting from septic complications when compared to wild-type animals. In the current study, we observed that in approximately half of the CSMG tested, exposure to propionate (C3) inhibited Ca^2+^ currents. The agonist-mediated responses were more robust at concentrations greater than 1 mM but inconsistent at lower ranges. Similar observations have been reported in two prevertebral (including CSMG) and two postvertebral sympathetic neuron types [24]. It is known that the cecum and colon contain higher concentrations of propionate than in plasma [19], though concentrations can increase significantly during fasting conditions [24]. Thus, the perforation of the bowel in the CLP model results in leakage of fecal material, along with bacteria, into the peritoneum. The added release of SCFA by bacteria would appear to activate both GPR receptors and likely suppress the sympathetic nerve activity, assuming that the receptors are located in the terminals. However, the role of SCFA in sepsis is not completely understood as these compounds can exert either pro- or anti-inflammatory actions [19].

The primary findings of the present investigation include (1) CSMG neurons isolated from septic rats exhibit a larger membrane surface area than controls; (2) sepsis induced a rightward shift of the NE concentration response curve, indicative of decreased NE potency in N-type Ca^2+^ channel modulation; (3) a subpopulation of CSMG neurons expresses MOR; and (4) both opioid pharmacological profiles and the propionate-mediated inhibition of Ca^2+^ currents are not altered by sepsis. From these results, it is clear that sepsis affects CSMG cell size and the adrenergic G protein signaling pathway such that Ca^2+^ entry via voltage-gated Ca^2+^ channels is altered. These morphological and pharmacological alterations, if analogous to the clinical setting, may help explain the detrimental effects of sepsis on the splanchnic circulation and the ensuing septic shock.
